# Retraction: miR-221 Promotes Tumorigenesis in Human Triple Negative Breast Cancer Cells

**DOI:** 10.1371/journal.pone.0175869

**Published:** 2017-04-10

**Authors:** The PLOS ONE Editors

The authors Min-Jean Yin and Rounak Nassirpour and a Pfizer representative contacted the editorial office to raise that there are image duplications in Figure 4B of this article. Pfizer has undertaken a review and deemed that important conclusions in the manuscript cannot be verified, as original images cannot be located.

In light of the concerns identified, the authors and the *PLOS ONE* Editors retract this article. We did not receive any response or comment from Pramod Mehta.

## References

[1] Nassirpour R, Mehta PP, Baxi SM, Yin M-J (2013) miR-221 Promotes Tumorigenesis in Human Triple Negative Breast Cancer Cells. PLoS ONE 8(4): e62170 doi:10.1371/journal.pone.0062170 23637992 10.1371/journal.pone.0062170 PMC3634767

